# Sixteen-Year Cohort of Liver Transplantation in the National Health System in Brazil: Analysis of Immunosuppression Maintenance Therapies

**DOI:** 10.3389/fphar.2020.572043

**Published:** 2020-10-06

**Authors:** Guilherme Fagundes Nascimento, Rosângela Maria Gomes, Juliana Alvares-Teodoro, Nélio Gomes Ribeiro, Mariângela Leal Cherchiglia, Charles Simão-Filho, Francisco Assis Acurcio, Tulio Tadeu Rocha Sarmento, Ludmila Peres Gargano, Augusto Afonso Guerra

**Affiliations:** ^1^Department of Social Pharmacy, College of Pharmacy, Federal University of Minas Gerais, Belo Horizonte, Brazil; ^2^SUS Collaborating Centre–Technology Assessment & Excellence in Health, College of Pharmacy, Federal University of Minas Gerais, Belo Horizonte, Brazil; ^3^Department of Preventive and Social Medicine, College of Medicine, Federal University of Minas Gerais, Belo Horizonte, Brazil; ^4^Department of Surgery, College of Medicine, Federal University of Minas Gerais, Belo Horizonte, Brazil

**Keywords:** graft survival, cyclosporine, tacrolimus, National Health Service, survival analysis, calcineurin inhibitor, immunosuppression, liver transplantation

## Abstract

**Objective:**

To evaluate factors related to liver graft survival with a focus on immunosuppressive schemes based on calcineurin inhibitors (tacrolimus or cyclosporine).

**Methodology:**

This study was carried out through an open cohort constructed by deterministic and probabilistic matching through three databases of the SUS with assessment of liver graft survival from 2000 to 2015 in Brazil. From this first cohort, a second cohort was constructed by pairing 1: 1 to more precisely assess the effect of the immunosuppressive scheme on graft survival. The Kaplan-Meier method and was used to estimate the probability of survival. Cox’s model of proportional risks was used to assess factors related to graft loss.

**Result:**

We found 12,687 patients in the Full cohort and 470 patients in the Matched cohort. The overall graft survival rates at 1, 5, 10, and 16 years were 72.6, 63.3, 52.8, and 45.3%, respectively. Patients younger had a longer graft survival than older ones. In the Full cohort, male patients had a higher survival rate than female ones. Therapeutic schemes based on tacrolimus were more prevalent and had a better survival rate when compared to schemes that used cyclosporine. Tacrolimus without association with antiproliferative agents or rapamycin inhibitors was the therapeutic scheme associated with greater survival rate in both cohorts (HR = 0.81, 95% CI = 0.72–0.91), (HR = 0.50, 95% CI = 0.30–0.85). In addition, white-skinned patients had longer survival rate in both cohorts (HR = 0.55, 95% CI = 0.50–0.61 and HR = 0.50, 95% CI = 0.34–0.75). On the other hand, patients who a greater time ratio without using an immunosuppressant had lower graft survival rate (HR = 6.46, 95% CI = 5.05–8.27 and HR = 6.57, 95% CI = 2.66–16.22).

**Conclusion:**

This 16-year cohort showed that the older age and the greater time ratio without using an immunosuppressant are risk factors for liver graft loss. White-skinned patients and tacrolimus-based regimens, especially tacrolimus without other immunosuppressants, are factors of better prognosis to the graft.

## Introduction

Currently, liver transplantation (LT) is well established in Brazil and worldwide. Since the first LT in 1963 in the United States, as well as the first operation in 1968 in Brazil, patients’ survival rate has progressively improved. This was possible due to advances in surgical methods, preservation solutions, early diagnosis, peritransplant intensive care, and immunosuppressive regimens (Starzl et al., 1968; Song et al., 2014; Burra et al., 2016).

The progress of immunosuppressive treatment was an important step towards success in LT. After the 1980s, calcineurin inhibitors (CIs) became the standard for immunosuppressives in LT. During this decade, cyclosporine was introduced as an immunosuppressive and there was a large increase in LT survival rates. In the following decade, tacrolimus, another CI, was used with patients who experienced rejection while taking cyclosporine. From that time on, tacrolimus became the standard drug for immunosuppression in LT (Korayem et al., 2017).

Brazil has one of the largest LT programs in the world, with public and free access, both for hospital treatment (surgery and hospital care) and outpatient immunosuppression with no need for co-payment (Guerra Junior et al., 2010; Meirelles Júnior et al., 2015). In 2016, Brazil was the third country in the world in the number of LTs performed, behind only the United States and China, which ranked first and second, respectively. However, in that same year, Brazil was the 21st in the number of liver transplants per million people in a ranking of 44 countries (Associação Brasileira de Transplante, 2018; Manyalich et al., 2018; Ming et al., 2019).

In the current transplant era the factors that impact survival rates depend on donor, recipient, perioperative and pos-operative parameters. Recipient age is a very important aspect of survival rate. Pediatric recipients have a better survival rate than the adult population and adults above 55 years have poor outcomes than youngers (Agopian et al., 2013). Previous study in Brazil shows the same results, recipient older than 65 have a shorter survival rate than youngers (Brandão et al., 2009). Gender influence in the outcome of LT is still controversial. Some works show females with a better survival rate and others show no sex effect in survival rate (Watt et al., 2010; Sarkar et al., 2015). Recipients white race seems to be a protective factor in LT (Roberts et al., 2004; Tae et al., 2007). Many other characteristics influence directly survival rate in LT like previous liver diagnosis— cancer has a poor survival rate—graft type, donor age, and number of transplants (Adam et al., 2018).

There is strong evidence that the use of tacrolimus improves the transplanted patient’s survival rate from one to three years when compared to cyclosporine (Haddad et al., 2006). When these immunosuppressants are compared over longer periods, evidence shows no difference in patients’ survival. However, it is known that cyclosporine presents a higher rate of graft loss than tacrolimus (Rodríguez-Perálvarez et al., 2017).

Our study’s goal was to analyze the data from the 12,687 LTs found in the cohort and identifying the factors, which influenced the long-term graft survival, as well as to compare the survival rate of the liver graft on the therapeutic regimens with tacrolimus and cyclosporine during an observation of up to sixteen years.

## Methods

### Study Design and Population

We conducted a historical open cohort study, including SUS patients undergoing LT in Brazil from January 2000 to December 2014 with sequence until December 2015. This cohort was obtained using deterministic and probabilistic pairing of three different SUS administrative databases: mortality information system (SIM), hospital information system (SIH/SUS), and high complexity procedure system (SIA/SUS). The date of the LT recorded in SIH was the first of the cohort. This same model was used in other studies published by our group in Brazil (Cherchiglia et al., 2007; Guerra et al., 2010; Gomes et al., 2016; Junior et al., 2018).

Initially, a cohort including all 12,687 liver transplanted patients found by the method was constructed in this period and this cohort was called Full cohort. Afterward, from the Full cohort, a new 1:1 Matched cohort was constructed using CI, sex of the recipient, age in years at the time of transplantation and the period of transplantation, named as Matched cohort. When more than one patient met the matching criteria, the allocation of pairs was done randomly. In the Matched cohort, the inclusion criterion was the age of the recipient over 18 years. Patients under 18 submitted to LT have peculiar characteristics that may interfere in the data analysis. The exclusion criteria were the absence of CI in the immunosuppressive scheme and the absence of registration of immunosuppressive treatment (Moreno and Berenguer, 2006; Watt et al., 2010; Daniel et al., 2017). We built the Matched cohort to reduce misleading factors and filter the role of immunosuppressants in graft survival.

The event used for survival analysis was the loss of the graft, defined as death or liver retransplantation. The right censoring was established on December 31, 2015. In Brazil, mortality reporting is mandatory, and continuous immunosuppressive treatment is given to SUS patients monthly.

**Figure 1:**
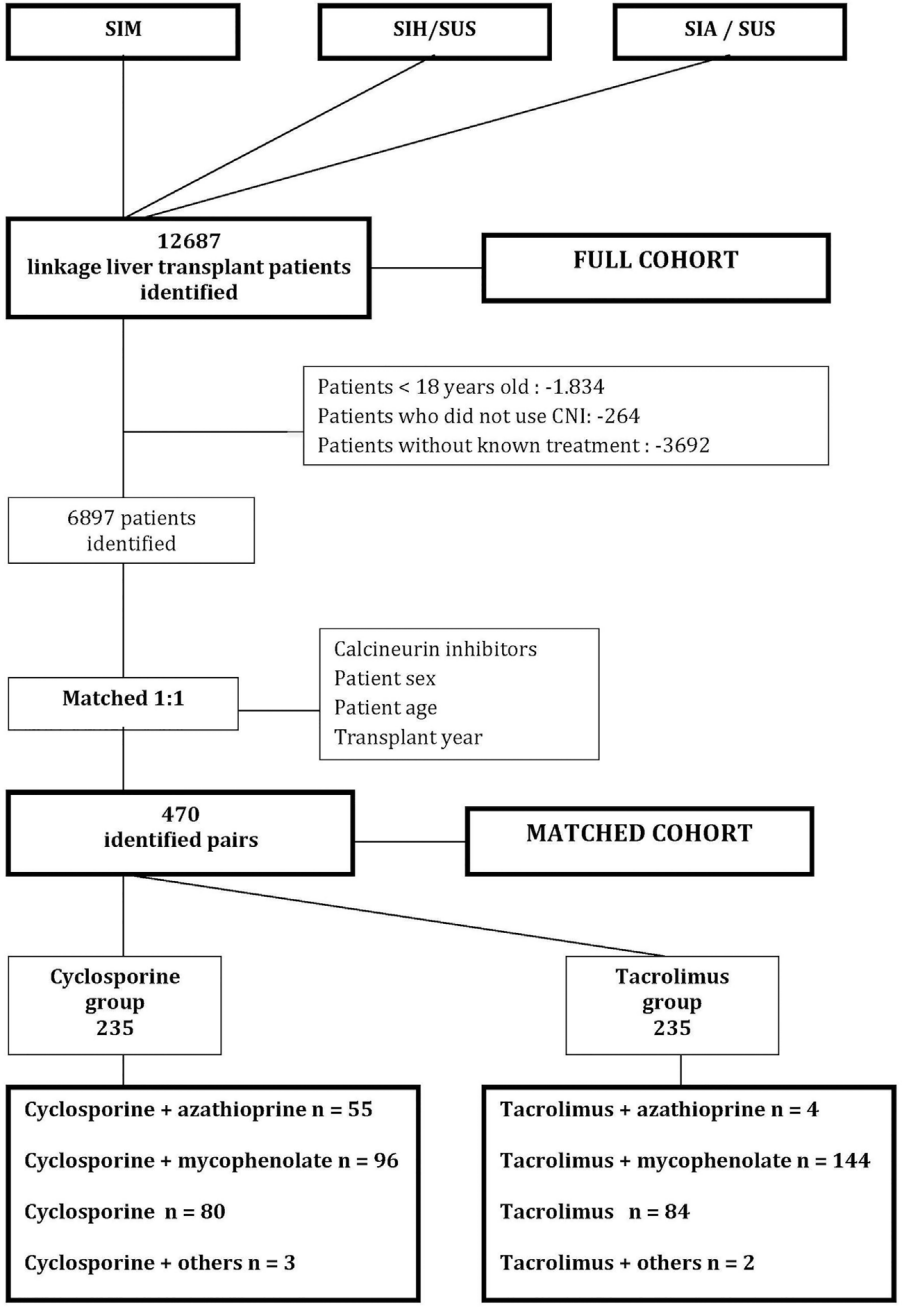
Cohort selection flowchart. CI, calcineurin inhibitor; SUS, Brazilian Unified Health System; SIM, Mortality Information System; SIH/SUS, Hospital Information System, SIA/SUS Outpatient Information System; Others: sirolimus or everolimus.

The graphical representation of the cohort selection flowchart can be seen in **Figure 1**. The patient’s therapeutic regimen was defined as the first time the patient was treated with the same regimen lasting at least 90 days. All therapeutic regimens were listed in the Full cohort. In the Matched cohort, the therapeutic regimens were stratified by the use of cyclosporine or tacrolimus associated or not with azathioprine or mycophenolate. The other associations to the CI used by the patients in the Matched cohort were included in the infrequent schemes category. It was considered that all patients of both cohorts used corticosteroids concomitantly, because it was not possible to track the dispensation of this drug by our database, since it is not a high-cost drug.

### Baseline Variables

The descriptive analysis of all variables used in the study was performed, with frequency distribution for categorical variables and a central tendency for continuous variables. The variables included therapeutic regimens; the geographical region of the hospital where the transplantation was performed; year of transplantation; the periods of transplantations, categorized in 2000 to 2003, 2004 to 2007, 2008 to 2011, and 2012 to 2014; the sex of the recipient; the age of the recipient at the time of transplantation; the age group of the recipient characterized by children and teenagers (under 18), adults (over 18 and under 65) and elders (over 65 years); the type of transplant (living or deceased donor); skin color, this variable was included in the database only in 2008; liver disease causes; the duration of liver diseases prior to the transplantation (greater or lesser than the median); the time ratio use of immunosuppressant; and the time ratio lacking immunosuppressants. The penultimate criterion was calculated dividing the number of months in which the medication was dispensed by the time of the patient in the cohort. The last criterion was calculated dividing the number of months without dispensing the medication by the time of the patient in the cohort.

### Statistical Analysis

The cumulative probability of graft survival over the 16-year cohort was evaluated by the Kaplan-Meier method and the survival curves according to immunosuppressants were compared by the log-rank test. Several factors that could affect the survival of the graft were initially evaluated by univariate analysis. The level of significance was set at 5%. The variables with a p-value less than 0.20 in the univariate analysis and those clinically relevant were included in the multivariate model. The relative risk of progression to the event adjusted for the multivariate model was calculated by the Cox proportional risk model and presented considering a 95% confidence interval (95% CI). The suitability of the multivariate model was measured by residue analysis. The statistical analysis was performed with the RStudio software, version 1.1.463, R Foundation for Statistical Computing.

### Ethics Statement

The research was approved by the Ethics Research Committee of Federal University of Minas Gerais (report no. 16334413.9.0000.5149). All subjects were anonymized; thus, the provision of informed consent was not required.

## Results

### Characteristics of the Patient

We identified 12,687 patients submitted to LT in the Full cohort and 470 patients in the Matched cohort, with an average follow-up of 34.5 and 61.1 months, respectively. The Full cohort included 859 elders (over 65 years), 9994 adults, and 1834 transplanted under 18 years of age, being the majority of patients male, with a male-female ratio of 1.89:1.

Most of the grafts came from deceased donors and the transplantations occurred predominantly in southeastern Brazil in both cohorts. White skin patients were majority among those who informed their skin color in both cohorts. Among the diagnosis of liver disease prior to the transplant, viral hepatitis corresponded to about 25% of them. All data with the characteristics of the patients are summarized in **Table 1**.

**Table 1:** Patient characteristics.

Parameters	Full cohort	Matched cohort	Cyclosporine	Tacrolimus
	n	n	n	n
	12,687	470	235	235
**Sex**				
Female	34,63%	22.12%	11.06%	11.06%
Male	65,37%	77.88%	38.94%	38.94%
**Age group**				
<18	14,46%	NA	NA	NA
18–65	78,77%	92.76%	46.38%	46.38%
>65	6,77%	7.24%	3.62%	3.62%
Skin color*				
Yellow	2,35%	2.55%	0.64%	1.91%
White	38,95%	57.44%	27.87%	29.57%
Brown	9,50%	5.32%	1.70%	3.62%
Black	1,70%	1.28%	0.85%	0.43%
Indigenous	0,01%	0,00%	0.00%	0.00%
Without register	47,49%	33.41%	18.94%	14.47%
**Regions of liver transplant**				
Southeast	56,99%	52.77%	23.83%	28.94%
South	23,53%	35.53%	25.53%	10.00%
Northeast	18,42%	11.49%	0.43%	11.06%
North	0,06%	0.00%	0.00%	0.00%
Midwest	1,01%	0.21%	0.21%	0.00%
**Eras of transplantation**				
2000–2003	13,83%	20.42%	10.21%	10.21%
2004–2007	19,41%	27.66%	13.83%	13.83%
2008–2011	34,31%	29.36%	14.68%	14.68%
2012–2014	32.45%	22.56%	11.28%	11.28%
**Liver Disease Before LT**				
≤Median	27,56%	28.94%	17.66%	11.28%
>Median	26,82%	33.62%	17.02%	16.60%
Without register	45,61%	37.45%	15.32%	22.13%
**Diagnosis prior to liver transplantantion**				
Alcoholic cirrhosis	9,89%	11.70%	5,53%	6.17%
Toxic liver disease	0,50%	0.42%	0,21%	0.21%
Cancer	0,28%	0.21%	0,21%	0.00%
Viral hepatitis	24,27%	27,66%	14.68%	12.98%
Others or indeterminant	65.07%	60.00%	29.36%	30.64%
**Type of transplant**				
Deceased donor	90,07%	97,88%	48,94%	48,94%
Living donor	9,93%	2.12%	1.06%	1.06%
**Events**				
Censoring	66.04%	78.72%	38.51%	40.21%
Death	27,85%	18,09%	10.00%	8.09%
Retransplant	6.11%	3.19%	1.49%	1.70%

^*Skin color was included in database only after 2008.^

### Graft Survival in Full Cohort

**Figure 2:**
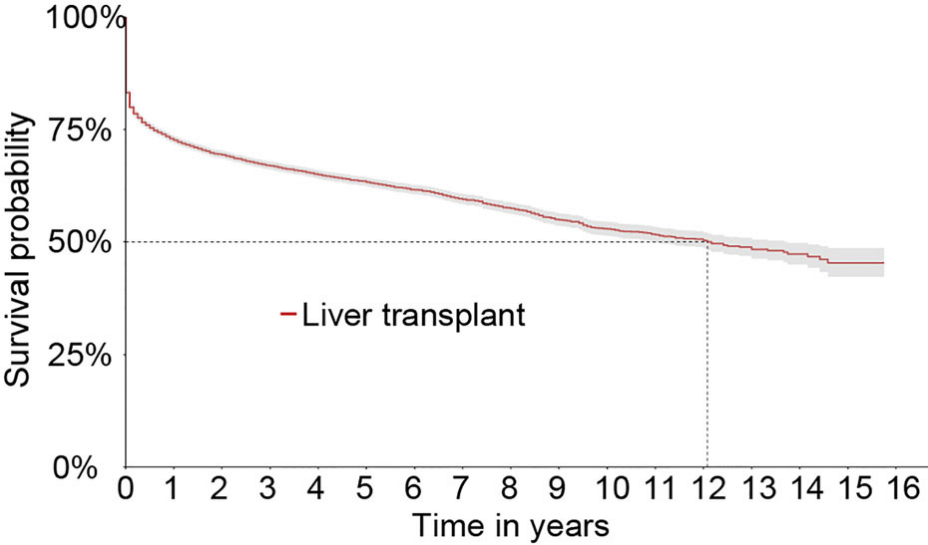
Kaplan-Meier survival curve of liver grafts from 2000 to 2015 in the Full cohort.

During the entire follow-up, 4,308 graft losses occurred (3,533 deaths and 775 retransplants) in the Full cohort. The estimated graft survival by the Kaplan-Meier method is shown in **Figure 2**. The general graft survival rates in 1, 5, 10, and 16 years were 72.6, 63.3, 52.8, and 45.3%, respectively.

**Figure 3:**
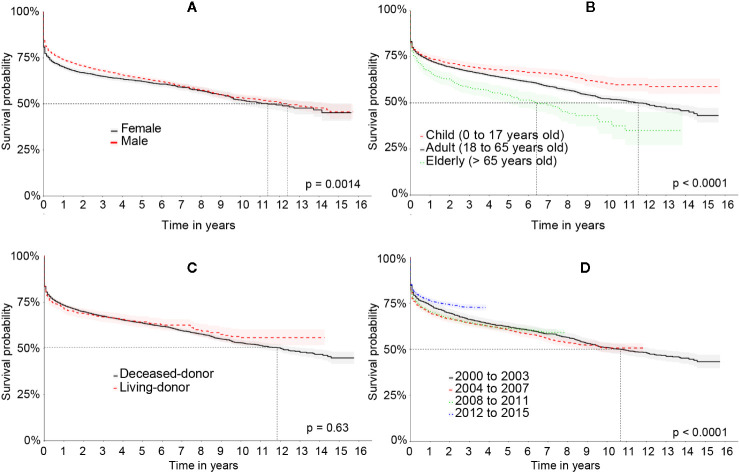
Kaplan-Meier’s survival curve of the liver graft in the Full cohort comparing: **(A)** patient’s sex, **(B)** age group, **(C)** type of transplant, and **(D)** period of transplant.

The survival rate of the graft was significantly higher in men when compared to women. The elders had a significantly worse graft survival rate compared to adults who, on the other hand, presented significantly worse results when compared to patients under 18 years old. The transplantation period from 2012 to 2014 had a significantly higher graft survival than the other ones. The length of time of liver disease prior to LT showed no difference in graft survival. The survival curves of the liver graft categorized by the characteristics of the patient can be seen in **Figure 3**.

### Graft’s Survival Rate in Matched Cohort

**Figure 4:**
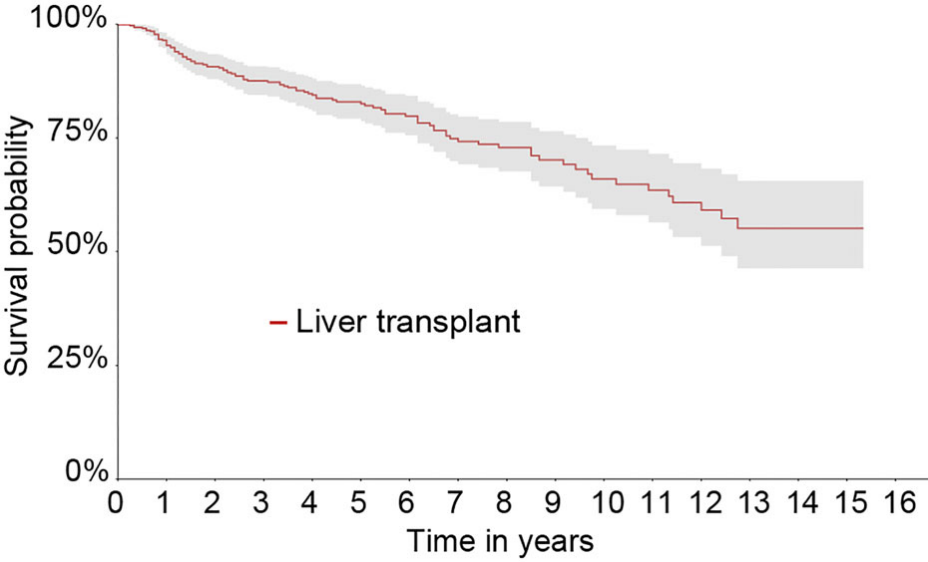
Kaplan-Meier survival curve of the liver graft from 2000 to 2015 in the Matched cohort.

One hundred graft losses (85 deaths and 15 retransplants) occurred in the Matched cohort during the 16 years of observation. The survival rates of the graft at 1, 5, 10, and 16 years were 95.3, 82.5, 66, and 55.1%, respectively. The estimated graft survival rate by the Kaplan-Meier method is shown in **Figure 4**.

**Figure 5:**
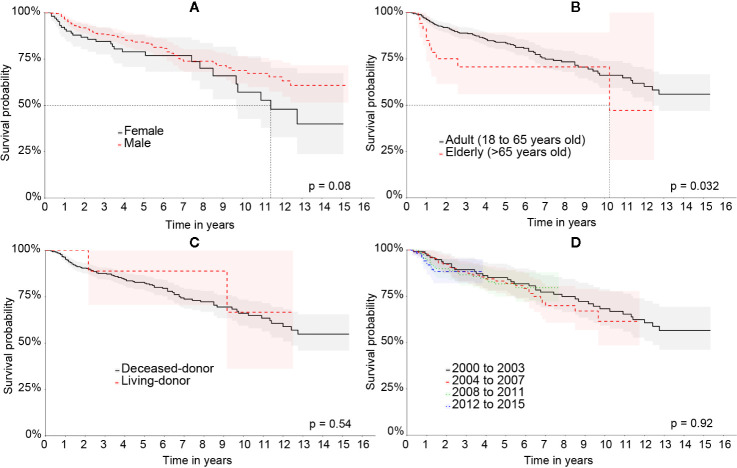
Kaplan-Meier’s liver graft survival curve in the Matched cohort comparing: **(A)** patient’s sex, **(B)** age group, **(C)** type of transplant, and **(D)** period of transplant.

Similarly to the Full cohort, the elderly also had significantly shorter graft survival when compared to the younger patients. Male patients had a higher survival than females ones, however, not statistically significant (p = 0.08). The transplantation region, the transplantation period, liver disease prior to LT, and the type of transplantation did not demonstrate statistically significant differences. The graft survival curves of the Matched cohort can be seen in **Figure 5**.

### Immunosuppressants

**Figure 6:**
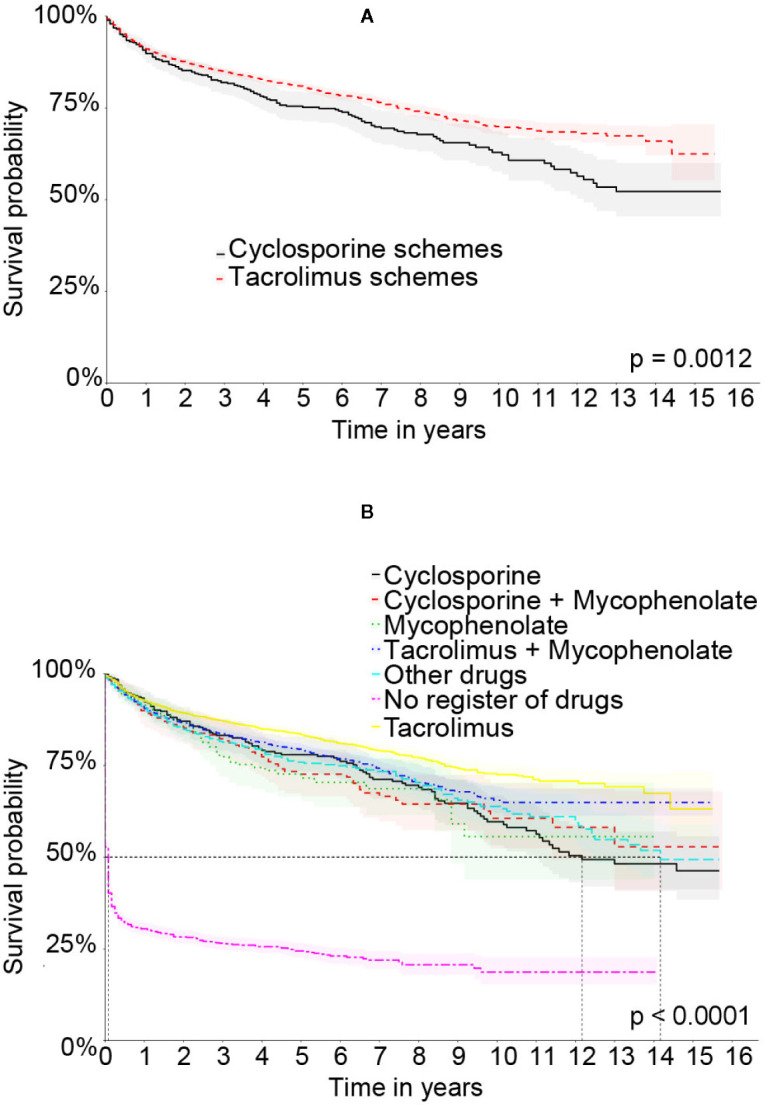
Kaplan-Meier’s liver graft survival curve in the Full cohort comparing therapeutic schemes: **(A)** Treatment based on tacrolimus or cyclosporine. **(B)** The specific therapeutic schemes.

The graft survival curve by Kaplan-Meier comparing the therapeutic schemes in the Full cohort can be seen in **Figure 6**. **Figure 6A** shows the curves with the therapeutic regimens based on cyclosporine and tacrolimus with a higher survival in the tacrolimus group with p < 0.001. **Figure 6B** shows the composition of the most frequent therapeutic regimens and evidences that the regimens that contained tacrolimus in their composition had a higher graft survival.

We found 28 different immunosuppression schemes in the Full cohort and the most frequent was tacrolimus associated with mycophenolate, followed by tacrolimus. The tacrolimus regimens were more frequent than the cyclosporine ones. All the schemes and their frequencies in both cohorts are described in **Table 2**.

**Table 2:** Full and Matched cohort immunosuppression therapy schemes.

Immunosupressant schemes	n	%	% cumulative
**Full cohort**			
Tacrolimus + mycophenolate	4060	48.95%	48.95%
Tacrolimus	2834	34.17%	83.12%
Cyclosporine	320	3.86%	86.98%
Mycophenolate	304	3.67%	90.64%
Cyclosporine + mycophenolate	269	3.24%	93.89%
Cyclosporine + azathioprine	231	2.79%	96.67%
Tacrolimus + azathioprine	131	1.58%	98.25%
Infrequent schemes	145	1.75%	100%
***Subtotal***	8,294	100.00%	
**Without register**	4,393	34.63%	
**Total - Full cohort**	12,687	100%	
**Matched cohort**			
Tacrolimus + mycophenolate	145	30.85%	30.85%
Cyclosporine + mycophenolate	96	20.43%	51.28%
Tacrolimus	84	17.87%	69.15%
Cyclosporine	81	17.23%	86.38%
Cyclosporine + azathioprine	55	11.70%	98.09%
Infrequent schemes	9	1.91%	100%
**Total - Matched cohort**	470	100%	

It was not identified the immunosuppressive treatment of 4,393 patients in the Full cohort. Out of these patients, 2,073 had graft loss before completing 30 days and 2,481 before completing the third month after transplantation. The majority of the patients who did not survive in this period of time were in this group, since there was a total of 2,669 graft losses by the third month after transplantation in the Full cohort.

**Figure 7:**
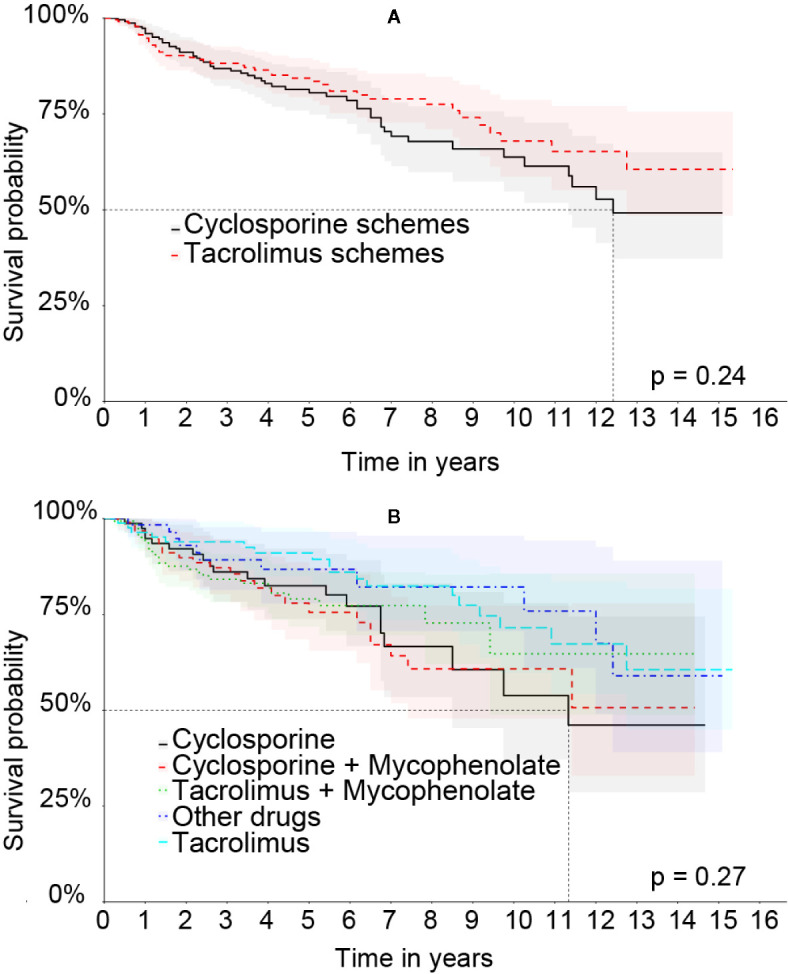
Kaplan-Meier’s liver graft survival curve in the Matched cohort comparing therapeutic regimens: **(A)** Treatment based on cyclosporine or tacrolimus. **(B)** The specific therapeutic schemes.

In the Matched cohort the graft survival curve by Kaplan-Meier comparing the therapeutic schemes can be seen in **Figure 7**. **Figure 7A** shows the survival curve combining the therapeutic regimens that used cyclosporine or tacrolimus. **Figure 7B** shows the survival curve of the most frequent therapeutic regimens. In both curves no statistically significant difference was observed.

### Univariate Analysis

Univariate analysis indicated lower risk of graft loss in male patients in the full (HR = 0.9, 95% CI = 0.84–0.97) and matched (HR = 0.68, 95% CI = 0.44–1.05) cohorts, however, not statistically significant in the latter (p = 0.08). Patients under 18 (HR = 0.85, 95% CI = 0.78–0.93) had longer survival rate than adults and elders. On the other hand, the elders had a lower survival rate than the others in the Full cohort (HR = 1.32, 95% CI = 1.18–1.48) and in the Matched cohort (HR = 2.02, 95% CI = 1.05–3.89). White skin patients had higher graft survival in the Full (HR = 0.41, 95% CI = 0.38–0.44) and matched (HR = 0.50, 95% CI = 0.34–0.75) cohorts. Patients who did not declare skin color had a lower survival compared to the others in the Full cohort (HR = 3.06, 95% CI = 2.87–3.26) and in the Matched cohort (HR = 2.01, 95% CI = 1.36–2.97). A higher proportion of tacrolimus use was a factor of higher survival in the full (HR = 0.49, 95% CI = 0.42–0.56) and matched (HR = 0.59, 95% CI = 0.35–0.98) cohorts. On the other hand, the higher the time ratio lacking the use of immunosuppressants, the lower the graft survival rate in both the full (HR = 4.04, 95% CI = 3.3–4.94) and matched (HR = 5.56, 95% CI = 2.35–13.39) cohorts. All factors of risk are described in **Table 3**.

**Table 3:** Univariate analysis–Risk factors associated with graft loss.

Risk factor	Full cohort		Matched cohort	
	HR (IC 95%)	p	HR (IC 95%)	p
**Male sex**	0.90 (0.84–0.97)	<0.01	0.68 (0.44–1.05)	0.08
**Age in years**	1.005 (1.003–1.006)	<0.01	1.02 (1.0–1.04)	0.1
**Age group**				
<18	0.85 (0.78–0.93)	<0.01	NA	
18–65	1.01 (0.93–1.08)	0.9	0.49 (0.26–0.95)	0.03
>65	1.32 (1.18–1.48)	<0.01	2.02 (1.05–3.89)	0.03
**Skin Color**				
Black	0.72 (0.56–0.94)	0.01	2.14 (0.68–6.75)	0.2
Brown	0.53 (0.47–0.60)	<0.01	0.82 (0.30–2.24)	0.7
Yellow	0.25 (0.17–0.37)	<0.01	0.81 (0.20–3.22)	0.8
White	0.41 (0.38–0.44)	<0.01	0.50 (0.34–0.75)	<0.01
Without register	3.06 (2.87–3.26)	<0.01	2.01 (1.36–2.97)	<0.01
**Regions of liver transplant**				
Southeast	1.03 (1.0–1.06)	1	0.96 (0.65–1.43)	0.8
South	1.19 (1.11–1.27)	<0.01	0.95 (0.63–1.45)	0.8
Northeast	0.83 (0.76–0.91)	<0.01	1.25 (0.68–2.3)	0.4
North	0.58 (0.30–1.13)	0.2	NA	NA
Midwest	0.31 (0.19–0.53)	<0.01	(0-∞)	0.7
**Immunosuppression scheme (basal)**				
Tacrolimus + mycophenolate	0.34 (0.31–0.36)	<0.01	1.13 (0.73–1.76)	0.6
Tacrolimus	0.31 (0.28–0.34)	<0.01	0.67 (0.39–1.1)	0.1
Cyclosporine	0.66 (0.55–0.80)	<0.01	1.24 (0.76–2.02)	0.4
Mycophenolate	0.53 (0.42–0.68)	<0.01	NA	NA
Cyclosporine + mycophenolate	0.59 (0.47–0.75)	<0.01	1.36 (0.85–2.16)	0.2
Cyclosporine + azathioprine	0.57 (0.45–0.73)	<0.01	0.82 (0.44–1.54)	0.5
Infrequent schemes	0.59 (0.50–0.69)	<0.01	0.71 (0.38–1.34)	0.2
Without register	10.6 (9.91–11.35)	<0.01	NA	NA
**Time ratio use of immunosupressor**				
Cyclosporine	1.14 (0.94–1.38)	0.2	1.03 (0.64–1.66)	0.9
Azathioprine	1.6 (1.12–2.28)	<0.01	0.96 (0.32–2.82)	0.9
Mycophenolate	1.05 (0.92–1.2)	0.4	1.08 (0.64–1.84)	0.8
Tacrolimus	0.49 (0.42–0.56)	<0.01	0.59 (0.35–0.98)	0.04
Everolimus	0.27 (0.05–1.45)	0.1	(0-∞)	0.7
Sirolimus	1.79 (1.08–2.96)	0.02	4.24 (0.63–28.9)	0.1
**Time ratio lacking immunosupressant**	4.04 (3.3–4.94)	<0.01	5.6 (2.35–13.39)	<0.01
**Type of transplant**				
Living donor	0.98 (0.88–1.08)	0.7	0.65 (0.16–2.62)	0.5
**Diagnosis prior to liver transplantantion**				
Alcoholic cirrhosis	0.91 (0.82–1.01)	0.07	1.05 (0.55–1.95)	0.9
Toxic liver disease	1.05 (0.68–1.61)	0.8	(0-∞)	0.6
Cancer	1.17 (0.68–2.02)	0.6	(0-∞)	0.8
Viral hepatitis	1.11 (0.69–1.76)	0.7	1.01 (0.70–1.76)	0.7
Others or indeterminate	1.01 (0.95–1.08)	0.7	0.92 (0.60–1.40)	0.7
**Transplant era**				
2000–2003	1.04 (0.96–1.13)	0.4	0.88 (0.55–1.40)	0.6
2004–2007	1.13 (1.05–1.21)	<0.01	1.09 (0.71–1.68)	0.7
2008–2011	1.11 (1.04–1.18)	<0.01	0.97 (0.60–1.57)	0.9
2012–2014	0.76 (0.71–0.82)	<0.01	1.15 (0.59–2.24)	0.7
**Year of hospitalization (t0)**	0.98 (0.97–0.99)	<0.01	1.02 (0.96–1.08)	0.5
**Liver disease before LT**				
>Median	1.06 (0.98–1.15)	0.1	0.94 (0.57–1.55)	0.8

∞ - Infinity symbol.

**Table 4:** Multivariate analysis–Risk factors associated with graft loss.

Risk factor	HR (IC 95%)	p
**Full cohort**		
Transplant period from 2012 to 2014	0.74 (0.64–0.85)	<0.01
Time ratio lacking immunosupressor	6.46 (5.05–8.27)	<0.01
Age (years)	1.01 (1.01–1.02)	<0.01
Ratio azathioprine use over time	2.55 (1.72–3.80)	<0.01
Ratio mycophenolate use over time	1.70 (1.41–2.04)	<0.01
Tacrolimus	0.81 (0.72–0.91)	<0.01
Yellow skin	0.33 (0.21–0.50)	<0.01
White skin	0.55 (0.50–0.61)	<0.01
**Matched cohort**		
Time ratio lacking immunosupressor	6.57 (2.66–16.22)	<0.01
Tacrolimus	0.50 (0.30–0.85)	0.01
White skin	0.50 (0.34–0.75)	<0.01

### Multivariate Analysis

Multivariate analysis revealed that the following variables in the Full cohort were associated with a higher risk of graft loss: time ratio of azathioprine use (HR = 2.55; 95% CI = 1.72–3.80); time ratio of mycophenolate use (HR = 1.70; 95% CI = 1.41–2.04), time ratio lacking the use of immunosuppressants (HR = 6.46, 95% CI = 5.05–8.27) and age in years at the time of LT (HR = 1.01, 95% CI = 1.01–1.02). On the other hand, white skin patients (HR = 0.55, 95% CI = 0.50–0.61); yellow skin patients (HR = 0.33, 95% CI = 0.21–0.50); those who used tacrolimus (HR = 0.81, 95% CI = 0.72–0.91) and those who performed the transplantation between 2012 and 2014 (HR = 0.74, 95% CI = 0.64–0.85) had a lower risk of graft loss. Multivariate analysis in the Matched cohort showed these as graft protection factors: white skin (HR = 0.50, 95% CI = 0.34–0.75) and tacrolimus scheme (HR = 0.50, 95% CI = 0.30–0.85). On the other hand, graft loss in the Matched cohort was related to the time ratio lacking immunosuppressant use (HR = 6.57, 95% CI = 2.66–16.22). All factors of risk are described in **Table 4**.

## Discussion

Brazil is one of the world’s largest LT performing countries and has one of the largest free public assistance programs in LT. This study is the largest cohort in Latin America which evaluated liver graft survival rate due to the immunosuppressive scheme, among other factors.

The Brazilian Unified Health System (SUS) is responsible for about 95% of LTs performed in the country. The guidelines of the Ministry of Health for Immunosuppression of Maintenance in Liver Transplantation in Adults or Children standardized behaviors for the LT treatment. These guidelines were created to ensure the best possible treatment in the Brazilian context, with the resources available in SUS. As in the rest of the world, the Brazilian guidelines chose CI as the main maintenance immunosuppressant after LT. Tacrolimus is usually the first option. CI can be used in monotherapy or with an antiproliferative agent (azathioprine or mycophenolate) or with everolimus, a rapamycin receptor inhibitor. These drugs can also be combined in maintenance treatment, depending on the patient’s characteristics. Generally, all immunosuppression schemes start in conjunction with the corticosteroid. Another inhibitor of rapamycin receptor, sirolimus, previously used in LT was excluded in the last Brazilian guideline. The induction of immunosuppression is done with methylprednisolone and/or thymoglobulin and/or basiliximab (Ministério da Saúde, 2016).

This cohort, despite not including all patients who underwent LT in Brazil in the period analyzed, presents a great representativity of the group. Patients who underwent LT outside SUS were not part of this cohort and there were also patient losses through the cohort construction method.

The graft survival in the Full cohort was different from the one shown in the Brazilian Registry of Transplants from the Brazilian Organ Transplantation Society (ABTO). The Brazilian Transplant Registry only included data from transplant teams that voluntarily reported 100% of their results in its analysis, but in 2018, only 55% of the teams met this requirement (Associação Brasileira de Transplante, 2018). Thus, bad performances of some teams may have been omitted. In our study, differently, the mortality was evaluated through SIM, where the mandatory mortality records are and the performance of a retransplant was surely seen by SIH. Even though, in the first year, the graft survival rate was similar both in ABTO (74%) and our study (72.6%), they differed in following years. In the fifth year after transplantation the graft survival rate was 67% according to ABTO and 63.3% in the Full cohort. In the ninth year, the maximum ABTO evaluation of survival rate was 63% compared to the 54.9% in the Full cohort. Information on liver graft survival in Brazil, up to then, was scarce and fragmented (Brandão et al., 2009). This study showed a long and reliable observation of liver graft survival.

The best results in the Matched cohort occurred by including only those patients who used CI for at least 3 months. Therefore, the patients with early mortality or retransplantation were excluded from the Matched cohort. Of the 12,687 patients in the Full cohort, 2,134 did not complete one month of graft survival and 2,669 had a graft loss before reaching the third month after transplantation, which corresponds to 21.3% of the patients in the cohort. This portrays a high mortality rate on the first 90 days following surgery.

This study results fall short when compared to those published from American and European cohorts, especially in the first 5 years. Since the 2000s, the adult liver graft survival rate when coming from a deceased donor in Europe in 1, 5, and 10 years was 80, 67, and 54%, respectively, and in the United States was 85, 75, and 55%, respectively (Adam et al., 2012; Berg, 2016).

The great difference of hepatic graft’s survival rate in the Full cohort compared to the rate of Americans and Europeans occurred especially due to the low survival rate in the first year and particularly in the first months after transplantation. It was not possible to evaluate the severity criteria of the patients, since we did not have this information in the database. However, this finding suggests the need to carefully examine the surgical procedure and post-transplant intensive care in order to discover the cause of the high rate of graft loss during this period.

Tacrolimus-based treatment regimens represented almost 85% of the treatments in the Full cohort and another 12% of the regimens were based on cyclosporine, the rest being the regimens that did not use CI. The frequency of tacrolimus usage is close to the one found in literature (Burra et al., 2016).

Analyzing Kaplan-Meier’s curves, it is possible to observe a higher graft survival rate on patients under tacrolimus-based therapeutic regimens when compared to those based on cyclosporine in the Full cohort. This finding is similar to that one of another cohort (Jonas et al., 2005) and the finding of Rodriguez’s meta-analysis (Rodríguez-Perálvarez et al., 2017). In multivariate analysis in the Full cohort, a higher proportion of azathioprine or mycophenolate use was related to a worse prognosis for the graft.

The Matched cohort was constructed to reduce bias in the interpretation of liver graft survival analysis specifically related to the immunosuppressive therapeutic scheme based on CI. Although the Kaplan-Meier curve showed no statistically significant difference in this cohort, possibly due sample number, the multivariate analysis in both cohorts showed that the tacrolimus therapeutic scheme was the only one related to the best graft survival rate. It is worth reinforcing that it was not possible to analyze the concomitant use of corticoids for any of the groups, nor the serum level of the CI, nor the severity of the patients in each therapeutic scheme. On the other hand, as expected, the time ratio lacking immunosuppressant was the most significant risk factor for graft loss in both cohorts. The lack of use of immunosuppressant, measured, in this case, by the absence of outpatient delivery of the drug, reflects the non-adherence to treatment or prolonged hospitalization of the patient in the period.

These findings resulted from the survival rate curve and multivariate analysis suggest that immunosuppression in LT with tacrolimus without antiproliferative agents is the most effective therapeutic scheme. A previous study indicated that tacrolimus in monotherapy or associated with corticosteroids is as effective as tacrolimus associated with antiproliferative agents (Lan et al., 2014). However, another interpretation would be that less severe patient cases did not need an association of immunosuppressants. Therefore, a better outcome could be justified.

A limiting factor in the interpretation of the graft survival’s data and its association with immunosuppressants was the absence of records of immunosuppressive therapy among 4,393 patients in the Full cohort. The main causes of non-registration were the death of the patient before the outpatient delivery of the drug, failure in registration, failure in deterministic pairing and dispensation of the drug by private health plan or private consultations. We faced this limitation and reduce potential bias by paired therapeutic schemes in the Matched cohort.

Data from the current study reaffirm that age is an independent risk factor for liver graft survival which shows better survival rates for younger patients and worse rates especially for those over 65 years old (Jain et al., 2000). Unlike other studies, this one showed that male patients had a better graft survival rate when compared to female patients in the Full cohort by the Kaplan-Meier method (Brandão et al., 2009; Watt et al., 2010; Fayek et al., 2016).

Other relevant factors related to improved survival of the liver graft were the period of LT between 2012 and 2014 and the yellow or white skin patients. A variable skin color was included in the database only in 2008. Then, the patients who lose the graft before this time had not skin color declared. We understand that this is a limitation of the study but if we exclude data for the period up to 2008 it would be a much greater loss for the study. The white colored skin proved to be a protective factor of the graft in the Full and Matched cohort by multivariate analysis as previously indicated in a different studies (Nair et al., 2002; Ananthakrishnan and Saeian, 2008). It is known that people with white skin color in Brazil have, in general, better socio-educational conditions, which may justify their better survival rate (Bastos et al., 2008).

Through the database, it was possible to identify records of liver disease before transplantation in part of the patients, and from these records, we were able to infer the basic cause of transplantation for some patients. However, in the vast majority of cases, it was not possible to evaluate neither the initial diagnosis that led to the transplantation nor the cause of graft loss. The vast majority of patients had generic diagnoses such as liver failure or cirrhosis, according to the database.

Brazil has presented important advances in LT, progressively increasing the absolute number of transplants, the number of transplant teams and the number of states that perform the transplantation, therefore improving the access and treatment of the population with terminal liver disease (Brazilian Organ Transplantation Society (ABTO), 2018). However, information on the outcome and impact of LT was either non-existent or from a disconnected source due to Brazil’s reality.

Despite the limitations of both the method and the records, this study was able to connect important information regarding LT, through the joining of three important SUS databases, which may help to improve LT care.

## Conclusion

This study evaluated liver graft through up to 16 years of observation and showed a survival rate of 72% in the first year and a mortality rate of 21% in the first 3 months following transplantation. It was shown that the transplanted patients who had an immunosuppressive scheme based on tacrolimus had less graft losses than the group that used cyclosporine. In addition, patients who were immunosuppressed with tacrolimus and had no association with antiproliferative agents or mTOR had a higher graft survival rate when compared to all other immunosuppressive schemes. In this cohort, the age and the time ratio lacking immunosuppressant were risk factors for graft losses, while white skin and time, considering the most recent transplantation, were criteria of better prognosis regarding the graft.

## Data Availabilility Statement

The raw data supporting the conclusions of this article will be made available by the authors, without undue reservation, to any qualified researcher.

## Ethics Statement

The studies involving human participants were reviewed and approved by Ethics Research Committee of Federal University of Minas Gerais (Report No. 16334413.9.0000.5149). The ethics committee waived the requirement of written informed consent for participation.

## Author Contributions

FA, JA-T, MC, and CS-F contributed conception and design of the study and manuscript revision. RG and NR contributed to manuscript revision and analyzed the results. TS and LG prepared the figures and analyzed the results. GN and AG wrote the manuscript and contributed in all parts of the research. All authors contributed to the article and approved the submitted version.

## Conflict of Interest

The authors declare that the research was conducted in the absence of any commercial or financial relationships that could be construed as a potential conflict of interest.
